# Additive Manufactured Magnesium-Based Scaffolds for Tissue Engineering

**DOI:** 10.3390/ma15238693

**Published:** 2022-12-06

**Authors:** Iulian Antoniac, Veronica Manescu (Paltanea), Gheorghe Paltanea, Aurora Antoniac, Iosif Vasile Nemoianu, Mircea Ionut Petrescu, Horatiu Dura, Alin Danut Bodog

**Affiliations:** 1 Faculty of Material Science and Engineering, University Politehnica of Bucharest, 313 Splaiul Independentei, District 6, 060042 Bucharest, Romania; 2 Academy of Romanian Scientists, 54 Splaiul Independentei, 050094 Bucharest, Romania; 3 Faculty of Electrical Engineering, University Politehnica of Bucharest, 313 Splaiul Independentei, District 6, 060042 Bucharest, Romania; 4 Faculty of Medicine, Lucian Blaga University of Sibiu, 550169 Sibiu, Romania; 5 Faculty of Medicine and Pharmacy, University of Oradea, 10 P-ta 1 December Street, 410073 Oradea, Romania

**Keywords:** Mg-based scaffolds, tissue engineering, additive manufacturing, bone defect treatment, regenerative medicine, bioresorbable implants, computer-aided design

## Abstract

Additive manufacturing (AM) is an important technology that led to a high evolution in the manufacture of personalized implants adapted to the anatomical requirements of patients. Due to a worldwide graft shortage, synthetic scaffolds must be developed. Regarding this aspect, biodegradable materials such as magnesium and its alloys are a possible solution because the second surgery for implant removal is eliminated. Magnesium (Mg) exhibits mechanical properties, which are similar to human bone, biodegradability in human fluids, high biocompatibility, and increased ability to stimulate new bone formation. A current research trend consists of Mg-based scaffold design and manufacture using AM technologies. This review presents the importance of biodegradable implants in treating bone defects, the most used AM methods to produce Mg scaffolds based on powder metallurgy, AM-manufactured implants properties, and in vitro and in vivo analysis. Scaffold properties such as biodegradation, densification, mechanical properties, microstructure, and biocompatibility are presented with examples extracted from the recent literature. The challenges for AM-produced Mg implants by taking into account the available literature are also discussed.

## 1. Introduction

Treatment of large bone defects is a big challenge in orthopedy. Due to an increased old age population, skeletal deformities, and different trauma accidents, high demand for bone implants is foreseen [1]. Large bone defects have a detrimental influence on the patient life quality. It is well known that bone exhibits self-healing abilities, but large bony defects, if left untreated, cannot heal by themselves. The bone graft is considered a first-line solution; every year, more than 2 million surgical interventions are carried out, so a shortage of natural bone grafts is expected [2]. The clinically used bone grafts are allografts, autografts, and xenografts. Autografts are the safest solution because the disease transmission risk and foreign body responses are drastically diminished. Unfortunately, these surgical interventions are characterized by drawbacks such as donor-site morbidity and the need for many operations in order to restore the defect [3].

The concept of synthetic bone is a valuable resource, and more and more scientists want to develop synthetic materials suitable for bone replacement [4,5]. An ideal bone substituent must exhibit a high biocompatibility rate, mechanical properties close to the ones of natural bone, avoidance of the stress shielding effect, a structure with interconnected pores to permit bony ingrowth, good biodegradability, and secondary products, which help the human body to heal [6,7]. It is challenging to develop a good bone substitute that can be used in clinical applications [8,9].

**Figure 1:**
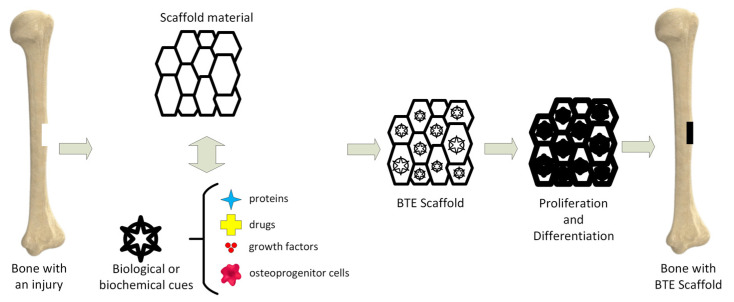
Bone tissue engineering (BTE) principle.

Tissue engineering (TE) is an important science branch based on scaffolds’ use to provide structural support to newly formed soft or hard tissues (Figure 1) [10]. Scaffolds are seen as temporary templates, which facilitate nutrient exchange between the human tissues and biomaterial, diffusion of oxygen, and cells’ metabolic waste elimination. These structures are adequate for the extracellular matrix (ECM) development in the affected area. They can be custom-designed to fit every patient’s anatomy through computer-aided designing programs. The computational modeling provides the scaffold’s pore size, shape, and geometrical dimensions. The designed scaffold can be materialized by additive manufacturing (AM) technology.

Scaffolds can be made from metals, ceramics, and polymers (Table 1) [11,12]. Metals exhibit high mechanical strength and fracture toughness and are suitable for load-bearing applications such as bone plates and screws, artificial joints, dental root implants, and tissue engineering scaffolds [13]. The most used biomaterials in orthopedy are cobalt–chromium-based alloys, titanium, tantalum, nitinol, and stainless steel [14]. These inert material implants are characterized by metal ions’ release or debris formation, which could have a toxic and carcinogenic effect during the material degradation process and can cause foreign body reactions and different inflammatory effects [15]. Another major complication is the stress shielding effect and the need for a second surgical intervention to remove the implant from the patient’s body if necessary. Metallic biomaterials with a good biodegradability rate have high potential because their released biodegradation products are biocompatible, and they can be metabolized in the human body, resulting in an increased implant integration process. Polymer-based materials can be easily functionalized with different organic or inorganic molecules, exhibit high design flexibility, and are usually biodegradable [16]. Unfortunately, polymers have low strength and hydrophilicity, and the apparition of aseptic inflammation at the implantation site limits their application in tissue engineering for bone regeneration [17,18]. Ceramics are highly biocompatible and bioactive but are brittle, making their application impossible in load-bearing zones. Due to their increased osteoconductivity, ceramic biomaterials can be combined with biodegradable metals [19].

Some important materials used for bone tissue engineering and their characteristics are presented in Table 1.

## 2. Biodegradable Magnesium Alloys

In biomedical research, the most used biodegradable metals are iron (Fe), zinc (Zn), and magnesium (Mg). Iron is an essential chemical element in the human body and is characterized by good mechanical properties, high biocompatibility, and a low degradation rate [34]. Its high elastic modulus is directly linked to a high radial strength. The degradation rate of iron is too low for it to be used in the tissue engineering domain at a large scale. Supplementary studies must be conducted to obtain a satisfactory corrosion rate, and Fe material properties must be tuned for it to be used in biomedical applications [35]. Zinc is useful for basic biological functions such as nucleic acid metabolism, deoxyribonucleic acid (DNA) synthesis, enzymic reactions, and apoptosis regulation [36,37]. Zinc can be found in the skin, liver, bone, and muscles. The Zn corrosion rate can be controlled through different forming processes or alloying with other materials. It was noticed that the Zn biodegradability rate is much more reduced in comparison with iron, and it can be considered a suitable material for TE. The main disadvantages of Zn are poor strength and ductility, which restrict the manufacture of implants for load-bearing zones [11].

**Figure 2:**
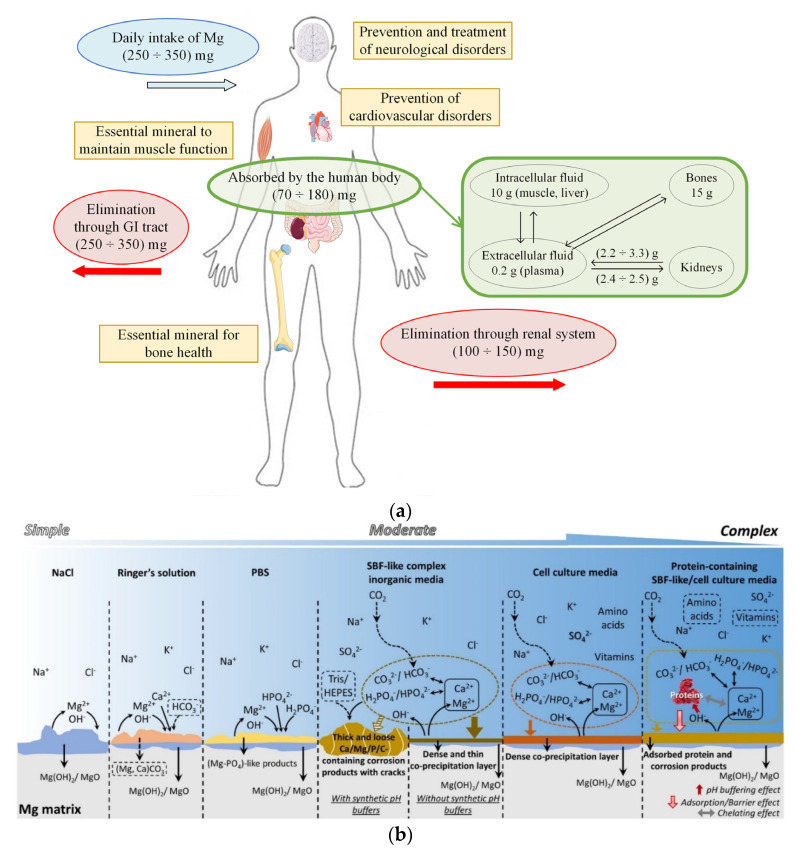
(**a**) Magnesium cycle in the human body and its beneficial effects on different biological functions; (**b**) corrosion behavior of Mg in the commonly used media (figure is licensed under CC−BY 4.0) [46].

Magnesium is a biodegradable metal that exhibits important properties and exceptional performance [38,39]. In the case of a daily intake of 350 mg, 25 mg is deposited in the human body, with almost half deposited in the bones, and the excess quantity is excreted in urine (Figure 2a) [40]. The density of Mg is about 1.7 g/cm^3^, and Young’s modulus is equal to 42 GPa, being very similar to those of human bone (density of 1.95 g/cm^3^ and Young’s modulus between 3 and 20 GPa) [41]. A rapid degradation process of Mg inside the human body causes hydrogen elimination and gas pockets’ apparition at the tissue–scaffold interface. A local alkalization process is present near the scaffold structure, and increased hydroxyl (OH^−^) ions can be observed [42,43]. These ions deteriorate the physiological microenvironment and can even generate an alkaline poisoning effect at a pH higher than 7.8. In order to improve the Mg properties, its degradation rate must be carefully checked and improved through surface treatment or alloying with chemical components leading to a decreased quantity of hydrogen gas and OH^−^ ions (Figure 2b). In this way, the human body can progressively adjust the biodegradation byproducts and heal using the beneficial effect of Mg^2+^ ions [44,45].

## 3. Magnesium-Based Scaffold Attributes

**Figure 3:**
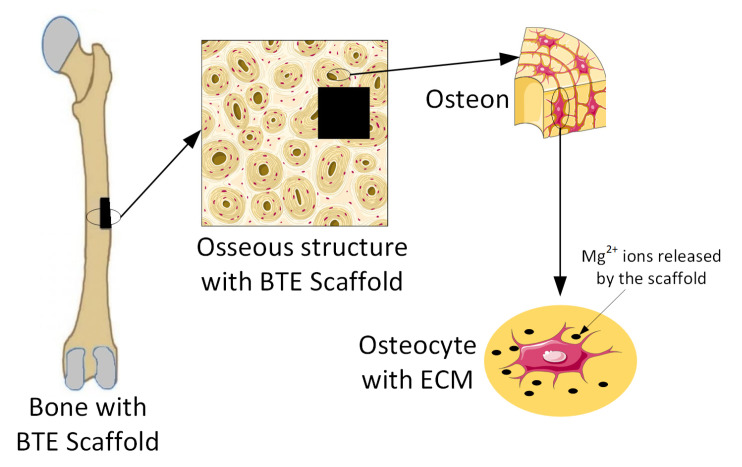
Release of Mg^2+^ ions from Mg-based implants into the ECM. Figure was generated using images assembled from Servier Medical Art, which are licensed under a Creative Commons Attribution 3.0 unported license (https://smart.servier.com, accessed on 15 September 2022).

Magnesium-based alloys are seen worldwide as innovative biomaterials, exhibiting good biodegradability and cytocompatibility when they are used for bone TE scaffolds [47,48]. Many in vitro or in vivo studies have proven that Mg implants help bone fracture healing (Figure 3) [1,49].

**Figure 4:**
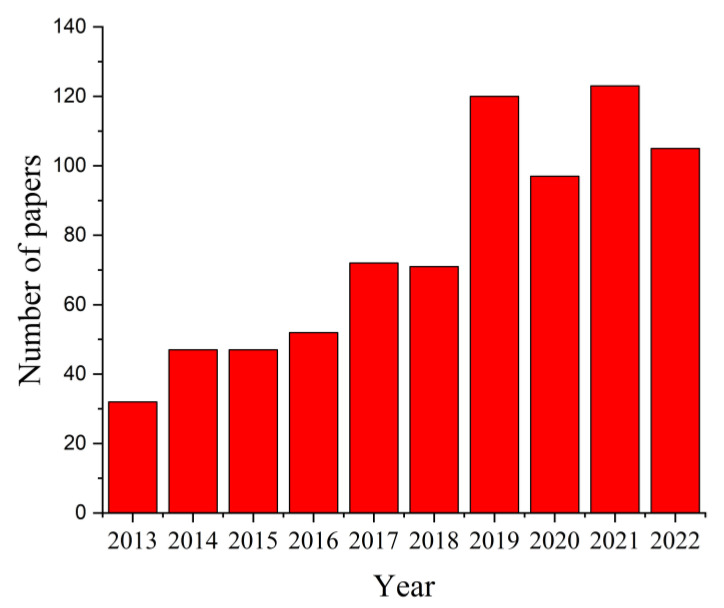
Statistical analysis results regarding the number of published papers between 2013 and 2022 that contain the search criterion “magnesium AND scaffolds”.

The search criterion “magnesium AND scaffolds” in the paper title OR abstract OR author keywords was used to search in the Science Citation Index Expanded and Emerging Sources Citation Index of Web of Science (WoS) Core Collection, considering ten years between 2013 and 2022, and 766 results were reported. The distribution of papers within this research field is presented in Figure 4. It can be noticed that this number increased from 32 articles in 2013 to 123 papers in 2021, a fact that supports the importance of this topic in worldwide studies. It was observed that in 2022 the number of papers investigating the Mg-based scaffolds was equal to 105.

The high-ranked journals that published papers in 2022 related to magnesium-based scaffolds and their impact factor are presented in Table 2.

An open porous structure based on Mg was developed by Yamada et al. [50], who used Mg-9Al-1Zn (AZ91) alloy. The manufactured scaffolds had a porosity of 99.7%, and the average pore size was between 0.3 and 4.5 mm. They used the infiltration casting method through polyurethane form replication. Later, Wen et al. [51] developed implants through powder metallurgy, combining Mg powder with carbamide and ammonium bicarbonate. Scaffolds with 50% porosity and fine pores with sizes ranging between 200 μm and 500 μm and good mechanical properties were obtained. Another investigated method for porous Mg-based implant manufacture is based on sodium chloride (NaCl) particles used as space holders [52]. The template replication method of infiltration casting can allow the manufacture of scaffolds characterized by the same pore strut and with different pore sizes. Jia et al. [53] have sintered NaCl particles into a template with an open porous structure, exhibiting a controlled pore size pattern, using NaCl particles and sintering necks [54]. In [55], the NaCl particles were substituted with titanium (Ti) particles, and the developed scaffold, after biological testing, proved to be a good regeneration process for the bone.

Many aspects must be considered when a scaffold is intended to be used in TE [56]. The most important scaffold attribute is biocompatibility because implants must be made from biocompatible materials with cellular scaffold components and endogenous host cells [57]. The encapsulated cells’ growth, migration, and differentiation are usually investigated using in vitro tests. Another important attribute of the scaffold is porosity. Pore size affects the scaffold porosity, its mechanical properties, and its degradation rate [58,59]. An ideal scaffold must have high pore interconnectivity [60]. An optimized pore size greatly influences different tissue regeneration processes because the scaffold is a suitable environment for cell proliferation [61]. The scaffold interconnectivity is considered a key factor, which acts as a modulator for cell migration and adhesion, blood vessel formation within the central implant region, and circulation of nutrients and cellular waste [62]. Interconnectivity can be defined as the pore space connections of 3D porous structures. Additionally, implant geometry is directly linked to cell performance and function. An implant should biomimic the human body’s native structure. In order to provide cellular attachment, scaffolds are functionalized with cell-adhesive ligands, which become a bioactive place for cell alignment and help cells to have a proper morphology. Sometimes scaffolds can be used as a carrier for growth factors, proteins, or drugs [63]. They are manufactured from biodegradable materials to permit the cells to fabricate their own ECM, and during a certain time, the materials are to be entirely replaced by the host tissues and cells. The secondary products resulting from the degradation process must be non-cytotoxic and safely excreted from the human body [64]. A large surface-area-to-volume ratio is beneficial for increased cell density and cell migration or adhesion. Mechanical properties of the implant are a crucial factor in TE because a scaffold has to provide sufficient mechanical strength to have a good shape after the extrusion process and during in vitro tests. Implants must have similar mechanical properties to the host tissue, and many research groups have evidence of the importance of mechanosensitivity. This phenomenon has a significant influence on cell adhesion and differentiation, and a direct link between material stiffness and the cellular process is presented in [63].

Successful Mg-based scaffolds for TE engineering were manufactured by drilling in bulk Mg pieces [65], infiltration casting [66,67], additive manufacturing [68,69], and hot press sintering Mg fibers [70,71]. Additive manufacturing (AM) is adequate for pore strut-free design. Three-dimensional (3D) scaffolds exhibiting a regular pattern of pores with different pore sizes can be printed through this technology. So, as a direct consequence, the scaffold porosity can be rigorously controlled.

## 4. Magnesium-Based Scaffold Production by Additive Manufacturing

Additive manufacturing (AM) is a suitable method to produce scaffolds, which have complicated geometrical shapes and mimic the ECM structures very well. This technology involves a layer-by-layer manufacturing process using computer-aided design (CAD) structures [72,73]. The AM methods are classified based on the feedstock material type (wire or powder) and the heat sources (arc, laser, or electron beam). According to American Society for Testing and Materials (ASTM) Standard F2792, there are two important types of AM technologies: directed energy deposition (DED) and powder bed fusion (PBF) [74,75].

PBF is one of the most used methods for metallic scaffold manufacture. Selective laser melting (SLM) and electron-beam melting (EBM) are the main thermal energy sources, which melt and fuse the metal powders in layers placed on a powder bed to generate a solid pattern [76,77]. Firstly, one layer of metal powder is put on a building platform, and then it is melted to a computer-designed shape [78]. The building platform is placed at a pre-defined distance, and a piston is involved in spreading and melting the next layer of powder on the previous layers [79]. In this way, layer by layer, the desired shape is constructed [42]. The thickness of one individual layer is between 20 and 100 μm. Rapid cooling of the build chamber is ensured by the circulation of argon or nitrogen, which creates conditions for a high-purity material of the scaffold by minimizing the oxygen and hydrogen in the chamber atmosphere [77,78].

Conventional manufacturing technologies such as powder metallurgy, foaming, casting, sintering electrodeposition, or chemical vapor deposition cannot provide a scaffold structure with a uniform shape and homogenous placement of the pores. AM gives the advantage of personalized implant production, made after the patient’s anatomy and with a regular pore shape and dimensions. This way, high control of pore architecture is directly linked to good porosity, permeability, mechanical strength, and stiffness [35,80]. Interconnected porous Mg scaffolds are obtained through AM with personalized shapes and internal architecture [35,81]. Ng et al. [82] have reported the successful melting of a single Mg layer. Recently, many studies have investigated scaffold manufacture with the SLM process, selective laser sintering (SLS), and binder jetting. It can be concluded that AM is the best way to produce metallic scaffolds with desired geometrical shape, controlled pore architecture, good mechanical properties, and high biocompatibility.

The PBF process provides control over material distribution and phase composition. Marangoni convection occurs due to high-temperature gradients in melting points, leading to a homogenous alloying material. It is well known that the reduced precipitated phase of the amalgam matrix solution could decrease the deterioration process by diminishing the electrochemical cell coupling between the return leg and the matrix. Magnesium exhibits a heat capacity of 650 °C and a boiling point of 1091 °C, making laser shaping a difficult procedure. During this step, Mg is burned off and oxidized, at the same time losing its binding efficiency. An important disadvantage of AM consists of the surface quality, and post-processing steps must be applied. In addition, custom alloying of materials is impossible through this technology, and almost all of the AM procedures are mainly linked to a given limit of the fabricated sample sizes [83].

Table 3 presents the AM technology’s advantages and disadvantages for Mg-based scaffolds.

**Figure 5:**
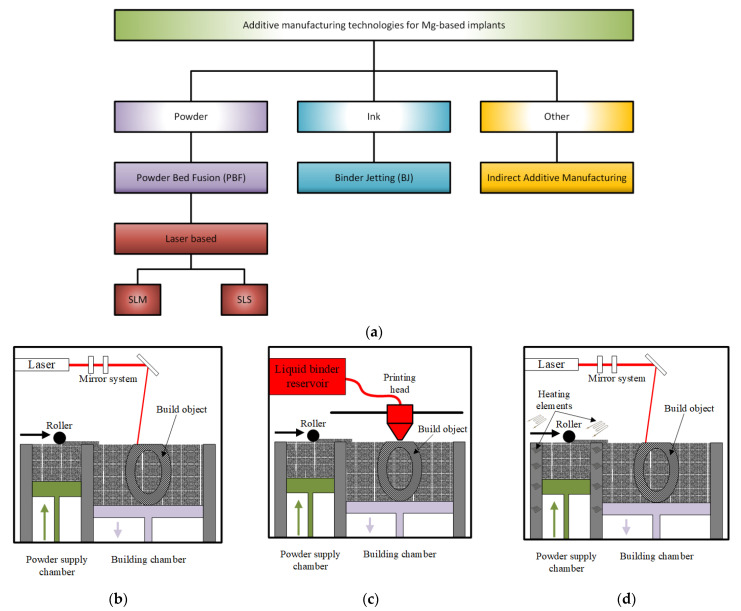
AM technologies used in the Mg-based medical implants’ manufacture: (**a**) schematical representation; (**b**) selective laser melting (SLM); (**c**) binder jetting; (**d**) selective laser sintering (SLS).

Figure 5 gives a schematical representation of the main additive manufacturing technologies involved in the Mg-based scaffolds’ manufacture.

### 4.1. Selective Laser Melting

SLM is included in PBF class methods. Its principle consists of melting and fusing the pre-spread powders layer by layer in a selected place based on a strong laser source and a CAD model, as mentioned above. The employed system comprises a laser, a construction station, a powder particle supply system that is automatized, dedicated software, and some important accessories (Figure 5b) [95]. A laser diffraction device made of a galvanometer and a flatter sector lens gives direction to the laser beam on the building table. SLM selectively melts the powder particles layer by layer to finish the printing of the desired component, which has 99.9% relative density [96]. The entire process is controlled through a dedicated program, which considers the powder input, the layering process, the scanning, the cooling and the heating steps, and the component construction. The main SLM phases can be synthesized as follows: firstly, a CAD model is created, and then it is divided into layers with a thickness between 20 and 100 μm; a substrate is leveled and secured on the build platform to prevent surface damage, a protective layer of inert gas is supplied. After that, a coating with powder material, whose thickness is equal to that of the thin designed layer, is applied. Scanning and handling of powder beds are carried out to build the part layer by layer. In the SLM process, a contour outline of the part geometry is generated, and the powder is melted inside this outline [97].

The SLM technique is characterized by inhibiting grain growth through rapid solidification that is obtained by involving fast heating and cooling cycles higher than 10^5^ K/s [98,99]. The segregation of the composition is reduced, having, as a result, a homogeneous microstructural architecture of the scaffold. Through this method are produced implants with high density, good mechanical properties, and degradation resistance [100]. During SLM, there are a high number of heating and cooling cycles that may have an unwanted effect consisting of a small heat-affected zone (HAZ), which grows around a melting pool. This phenomenon changes the material’s chemical composition and can have an important effect on physical properties.

Zumdick et al. [101] have analyzed the properties of Mg-4Y-3RE-0.5Zr (WE43) magnesium-based alloys produced through the SLM technique. A very fine grain structure with an average grain size of around 1 μm was observed. The samples had an ultimate tensile strength of 308 MPa and 12% elongation to failure. Bar et al. [102] have given evidence of an improved biodegradation property in the case of SLM-manufactured samples by comparing these with those made through the casting method. Li et al. [69] have fabricated topologically ordered porous scaffolds made from WE43 alloy with a diamond unit cell through the SLM process. The implant strut size was about 400 μm, the pore diameter was equal to 600 μm, and the material porosity was 67%. The mechanical properties of porous Mg-based alloys were in the range of those for the trabecular bone, with a Young’s modulus between 0.5 and 20 GPa. AM-produced scaffolds exhibited a biodegradation behavior characterized by 20% volume loss after 4 weeks. These scaffolds had a low toxicity level. The sample fatigue resistance of the AM-manufactured WE43 scaffold was reduced to about 0.2 σ_y_ [103]. It was noticed that the optimization of the topological design and laser processing parameters have a great influence on the scaffold microstructure.

Chen et al. [100] have made a binary Mg-Zn alloy through the SLM technique and found a homogenous grain structure with an average size of 15 μm. The precipitation of the MgZn phase and the rapid solidification process inhibited grain growth. Wei et al. [104] have studied the influence of Zn content in the Mg-Zn binary compound. They have found that 1 wt% of Zn has a beneficial effect on the mechanical properties, which were in the range of those measured for as-cast samples. The corrosion resistance of the binary Mg-Zn alloys is improved by adding aluminum (Al). In [104], the manufacture of parts from ternary Mg-6Al-1Zn alloy (AZ61), using the SLM process, was analyzed. At a laser input energy of 80 W, an equiaxed grain structure and a maximum microhardness of 93 HV were reported. Another study [105] investigated the effect of yttrium (Y) addition on the degradation behavior of magnesium and an increase in this parameter was found. Another modality to improve the Mg-Zn binary alloy corrosion resistance is hydroxyapatite (HAp) incorporation. Shuai et al. [106] have made Mg-3Zn/xHAp composite materials using the SLM method. The rapid solidification process prevented the HAp particles’ agglomeration, and a homogenous dispersion process was observed. When the hydroxyapatite percent increased, a structure with finer grains was obtained, leading to an increased corrosion resistance due to apatite coating. The material hardness becomes higher due to fine grain structure and second-phase strengthening.

Zhang et al. [107] have investigated the addition of Zr to binary Mg-Zn alloys, and they have found that by increasing the Zr content, the grain size is reduced, and the material exhibits a low degradation rate. Additionally, by modifying the laser intensity, a very good surface quality was noticed. By adding dysprosium (Dy), ternary alloys of Mg-Zn-Dy are obtained. They are characterized by low degradation and hydrogen evolution rate, small grain size, and homogenous microstructure [98]. The selected studies evidence that the SLM procedure leads to Mg-based scaffolds with high-quality microstructure, good mechanical properties, and high biodegradation performance.

### 4.2. Binder Jetting

This method is a two-step process. During the first stage, a metallic powder layer is deposited on the powder bed. The particles are bonded in a specified region through a chemical reaction or adhesion process (Figure 5c) [99]. In the case of chemical reactions, a solution, which is dropped on the specific bonding points, reacts with the powder particles [108,109]. For the adhesion process, a solid or liquid polymer is used to glue the particles in the desired shape layer [110,111]. Some technologies require a curing process to increase the green compound’s mechanical strength. After this step is finished, a self-supported structure results and must be extracted from the powder bed. The second step consists of a debinding process to remove the binder and unbonded powder and a post-treatment process, such as infiltration with different materials [112], sintering [113], and hot isostatic pressing [114].

Farag and Yun [115] have investigated the effect of gelatin addition, in an amount lower than 6 wt%, on the fabrication of magnesium phosphate-based scaffolds (MgP). The formation of a dense strut and an enhancement of the mechanical properties were observed. Furthermore, the MgP/gelatin scaffolds exhibit a hydrophilic behavior and a very good cell affinity. Meininger et al. [89] have manufactured, using the binder jetting method, strontium (Sr)-substituted Mg_3_(PO_4_)_2_ scaffolds. Good mechanical properties such as compression (36.7 MPa), bending (24.2 MPa), and tension (10.7 MPa) strength were experimentally determined. Water was used as a binder, and sintering and hardening processes were applied as post-treatments. In vitro tests showed a reduced release of magnesium ions and improved corrosion resistance. The microstructural analysis gives evidence of an interconnected topology characterized by a 20 μm average pore size. Salehi et al. [116] have shown how the capillarity-driven bridging phenomenon can be used for assembling powder particles in 3D structures. The method is based on magnesium oxide (MgO) film conversion on the outermost layer of Mg powder into an interparticle bridge. In this way, the capillary-mediated assembly of particles makes the use of the polymeric binder unnecessary. A scaffold with a constant composition was obtained.

The main drawbacks of the above-described method are sacrificial binder use and additional processes necessary to remove the binder. As the powder particles are glued together, the resulting green compounds exhibit low mechanical properties. Post-processing operations are mandatory for scaffold densification.

### 4.3. Selective Laser Sintering

This method is based on a bed of compacted powder particles, which are heated at a temperature close to the melting transition point (Figure 5d). The powder particles are bound together with the help of a laser beam that is traced over the bed surface. The laser draws different patterns onto the powder surface during the printing process. When the first layer is synthesized, the incorporated platform is lowered by 100–200 μm, and new fresh powder particles are spread using a roller. After each powder layer is finished, new layers must be heated over their crystallization temperature to be completely melted to ensure an adequate bonding between particles and to avoid the cooling of the previous layers. In this way, the deformation of the sintered layer is hindered (Figure 4d). The 3D objects are printed layer by layer, and they can be collected from the powder bed. Local thermal sintering of the particles is given by a high-power carbon dioxide (CO_2_) laser that melts the powder after scanning it in a given way. The fabrication chamber is sealed, and its temperature is kept under the melting point value [117].

Tsai et al. [118] have manufactured and analyzed 3D composite scaffolds made from magnesium–calcium silicate/poly-ε-caprolactone (Mg-CS/PCL). The composite powder of Mg-CS was incorporated into PCL, and the scaffolds were made based on the selective laser sintering method. The developed implants exhibited a high porosity grade and an interconnected design macropore structure. Good hydrophilic properties and degradation rates were noticed. In vitro analysis has shown high biocompatibility of the scaffolds, which enhanced human mesenchymal stem cells’ multiplication and adhesion with the help of released Mg^2+^ ions.

Selective laser sintering-manufactured scaffolds have a low value of density and poor mechanical properties because the partial melting process of the powder particles is linked to pore formation and struts inside the metallic material.

### 4.4. Indirect Additive Manufacturing

Magnesium scaffolds can also be manufactured through infiltration technology. A polymeric template, whose model is designed in CAD software, is made using the AM method. The template is infiltrated with a NaCl paste, and the polymer is removed later by heating at a specific temperature. The result consists of a negative NaCl template formation, in which liquid Mg is cast using applied pressure. Finally, the NaCl is dissolved, and only the Mg structure remains [119].

Based on this technology, Nguyen et al. [80] have developed an indirect solid free-form fabrication (SFF) process to manufacture topologically ordered Mg structures. It was shown, using different characterization devices, that the developed Mg-based structures were made with a high accuracy grade. Minor differences between CAD models in the interval of 2.5% and 8.33% were determined. A maximum of 6.1% reduction in the porosity of Mg implants was detected in the case of some structures by comparing them with the initial design. An average increase of 70% in surface area was seen, and it was concluded that the technology presented in the paper is reliable, simple, and safe for implant manufacture.

Lin et al. [120] have fabricated metallic hybrid composites by printing 3D CoCr scaffolds, in which they have infiltrated Mg-3Al-1Zn (AZ31) magnesium alloy based on a pressure-less infiltration technique. The degradation behavior of the scaffolds was investigated through the immersion method in Hanks’ solution. It was concluded that the degradation rate of the scaffolds is higher than in the case of AZ31 alloy due to galvanic corrosion effects. It was noticed that this phenomenon is strongly dependent on the surface area and composite interface. A reduced value for stiffness and strength is observed regarding the mechanical properties. The authors have concluded that parameters such as scanning speed and part geometries are essential and can significantly influence indirect AM process failure.

In [121] is reported a novel technique, which combines the salt-leaching method with AM. They have successfully modified the solvent and surfactant composition, so the salt-based paste is engineered from a rheological point of view and directly printed into grid-like structures. This way, porous scaffolds with controlled pore size and ordered geometry were made. It was concluded that the implant’s mechanical properties are a function of the material porosity and can be modified until a specific performance is obtained.

The infiltrating technology eliminates the use of Mg powder, and the explosion danger of the previously discussed methods is hindered. Unfortunately, using indirect additive manufacturing, the geometrical characteristics of struts and pores are related only to the macroscale, which in some cases is a drawback in medical applications, where an open porous structure is necessary.

## 5. Specific Properties of the Magnesium-Based Scaffolds

The design geometry and the scaffold microstructure have an important influence on the biological and mechanical properties of the magnesium-based scaffolds. Some important aspects, such as biodegradation, densification, mechanical properties, and biocompatibility, should be considered.

### 5.1. Biodegradation

The scaffold biodegradation depends on the design, material, and manufacturing process [122,123]. In [124], the corrosion behavior of pure Mg fabricated through the SLM procedure was investigated. It was concluded that the process parameters significantly influence the material porosity and corrosion rate. This last parameter was computed as a function of the material mass before and after the corrosion and of the immersion time.

It is well known that the Mg alloy corrosion can be diminished through grain refining treatments. Li et al. [69] manufactured scaffolds made from WE43 using the SLM technique. They obtained an enhanced biodegradation resistance of 0.17 mL/cm^2^ day by comparing this value with as-cast or as-extruded samples. He et al. [125] studied the degradation resistance of AZ61 alloy and observed that this factor was improved by rapid solidification through SLM. The laser power was increased, and enhanced microhardness and degradation rate were evidenced. It was concluded that this fact is due to the coarsened equiaxed grains and a reduced solution of aluminum in the magnesium matrix. Shuai et al. [126] fabricated Mg-6Zn-0.6Zr (ZK60) based on the SLM method. They increased the laser’s energy density, leading to grain refinement, homogenized microstructure, and rapid solidification during the SLM procedure. The material corrosion resistance was highly improved.

Magnesium-based alloys reinforced in bioceramic materials can exhibit good mechanical strength and corrosion resistance. Deng et al. [127] have prepared β-tricalcium phosphate (β-TCP) combined with ZK60 using the SLM method. Due to the rapid solidification process, a homogenous distribution of β-TCP placed along the grain boundaries of α-Mg was noticed. If rare-earth metal such as Nd is incorporated into Mg-based alloys via SLM technology, fine α-Mg grains and intermetallic phases are obtained. A surface layer promoted by Nd_2_O_3_ formation inhibited the degradation resistance of the composite material, and the neodymium (Nd)-induced honeycomb structure was evidenced [128].

Intermetallic phase apparition is considered an important factor that influences biodegradation property. Different grain sizes and intermetallic phase volume fractions were created by varying the Al concentration in Mg-3Zn-0.6Zr (ZK30) alloy using SLM. It was noticed that the fraction of the intermetallic phase and grain size refinement are enhanced by increasing the Al content. However, when the Al content is below 3 wt% due to the apparition of numerous grain boundaries, it is possible to passivate the material surface faster, and thus an increased corrosion resistance is obtained. A summary of different studies found in the literature is presented in Table 4.

### 5.2. Densification

The formation quality is described in the literature with the term densification, which also considers process parameter optimization. During the manufacturing procedure, processing pores can appear, which can be located inside the scaffold struts. They have a detrimental influence on the implant’s mechanical and biological properties. A relative density higher than 99.5% was obtained through SLM technology in the case of bulk Mg alloys [94]. Liu et al. [129] made porous Mg-Ca alloys through a laser additive manufacturing process. They noticed that the porosity and surface morphology are directly linked to the laser energy input. This parameter was varied between 875 J/mm^3^ and 1000 J/mm^3^, and the obtained porosity was between 18.48% and 24.60%. The microhardness of the porous Mg-Ca was superior to that of as-cast pure magnesium and was between 60HV and 68HV. The formation quality of the developed porous scaffold was about 81%. Shuai et al. [126] found that by increasing the laser energy density, the crystalline structure of Mg-Zn-Zr alloys successively changed from clustered finer dendrites to uniform equiaxed grains and coarsened equiaxed grains. An ideal structure without open pores and a relative density of 97.4% was obtained at a laser energy of 600 J/mm^3^. By increasing this factor to 750 J/mm^3^, the apparition of microcracks on the surface was observed. Li et al. [69] have manufactured from WE43 porous scaffolds exhibiting a diamond lattice. They have reported some geometrical discrepancies regarding the as-built strut size of 420 μm by comparing it with the designed value of 400 μm and an as-built porosity of 64%, slightly smaller than the design value of 67%. Qin et al. [130] have made a diamond lattice porous scaffold using Zn + WE43 powder. The relative density was about 99.7. Unfortunately, geometrical discrepancies were also reported in this case. The as-built strut size was 562 μm, much larger than the design value of 400 μm. The as-built geometrical porosity was about 45% to 67%, characteristic of the initial design. The main recent studies presented in the literature are synthesized in Table 5.

Usually, the properties of the PBF components depend on the input laser power, spot diameter, hatch spacing, scanning speed, and layer thickness [132]. By selecting the correct values for these parameters, high-density parts can be manufactured. The ideal energy density dependence on the parameters mentioned above is given in [94]. Lower values of the scanning rate increase the density of the component due to a longer interaction time between the laser beam and powder that determines a higher energy delivery rate to the powder layer. In the case of high scanning rates, the laser energy transferred to the powder layer is reduced, so a partial melting between powder particles appears, which leads to pore creation within the struts. For a proper energy value, the particles are fully melted and penetrate the spaces, and a thick part is obtained.

### 5.3. Mechanical Properties

Mechanical properties of the AM Mg-based implants include tensile and compressive strength, stiffness, durability, ductility, and hardness flexibility. The elastic modulus of the scaffold must have a similar value to that of cortical bone to avoid the stress shielding effect. The relative density of the implant is the most important factor, which affects Young’s modulus and failure strength considerably, according to Gibson and Ashby’s model [133].

σ_pl_ = 0.3 (ρ/ρ_s_)^1/2^ ρ_ys_, (1)

E = ρ(ρ_s_)^2^E_s_, (2)

where ρ is the density of the elastic substance, ρ_s_ is the solid material density (relative density), E is Young’s modulus, and σ_pl_ is the plastic failure strength. According to Equations (1) and (2), Young’s modulus decreases by increasing the material porosity. Grain refining can improve the implant’s mechanical properties, and it has an important influence on alloy microhardness.

Microhardness of Mg-Ca alloys depends on different parameters during the PBF process according to the Hall–Petch formula H = H_0_ + kd^−1/2^, in which H_0_ and k are constants that depend on the material crystalline lattice, d represents the grain size, and H depicts the sample hardness. The average value of the microhardness is between 60 HV and 68 HV [83]. For the PBF-produced Mg-Ca, a solid-dissolution of calcium (Ca) into the α-magnesium matrix is noticed. This solid solution strengthening can improve the hardness and strength of the alloy and is directly linked to the alloy’s higher value of microhardness. The dislocation density and lattice deformation are due to the rapid cooling rate and impact the microhardness value. For PBF Mg-Ca alloys, the grain size is between 5 and 30 μm, and in the case of pure magnesium, the same quantity has higher values between 300 and 500 μm. It is expected that the hardness of Mg-Ca materials is higher.

In the case of Mg-Zn-Zr alloys, the microhardness exponentially rises with the Zn content. An average value between 57.67 HV and 58.28 HV in the longitudinal section of Mg-5.2%Zn-0.3%Zr alloy was found, and regarding the Mg-15%Zn-0.3%Zr material, this value was between 75.51 HV and 80.23 HV. In the case of Mg-30%Zn-0.3%Zr, an interval of 106.75–109.36 HV was obtained [107]. According to the Hall–Petch equation H = H_0_ + kd^−1/2^, in which H is the sample hardness, H_0_ and k are material constants, and d is the grain size, it is expected that the grain size will decrease directly proportional to the Zn content.

The alloying elements influence the material grain size. In the case of Mg-Zn-Dy alloys, the grain size decreases directly proportional to the Dy content, so as a consequence, the microhardness increases [98]. The Dy content was varied from 0 to 5 wt%, and for the maximum wt%, a microhardness of 121.28 HV was obtained. This value is 1.38 times higher than that obtained for Mg-3Zn alloy.

By considering the solid solution strengthening theory, better mechanical strength results from a higher solid solubility. The atomic radii of Al and Zn are of the order of 0.11 nm, a value lower than the Mg radius of 0.13 nm. A lattice distortion occurs when these alloying elements are dissolved into the Mg matrix. In the case of binary alloys, Mg-Al, the variation of microhardness is directly proportional to the Al content in the α-Mg matrix [134]. The intermetallic compound in Mg-Al alloys is β-Mg17Al12, whose hardness can be reduced by applying SLM at the value of 150 ± 60 Hv since the hardness of α-Mg alloy is maintained at 126 ± 3 Hv. The SLM technique reduces the dispersion of the β-Mg17Al12 phase, but it leaves the hardness value of the alloy unchanged.

AM techniques that use the laser beam as a heat source are characterized through different thermal histories in the same sample (e.g., for SLM-processed materials, the microhardness measured at the center of the molten pool and the microhardness at the edge zone have different values) [135]. The presence of defects also has an essential influence on the material’s microhardness. A reduced value is obtained when the scaffolds exhibit a high porosity or cracks in the material microstructure. In the case of SLM technology, the rapid solidification process has an important effect on the alloy microstructure and the solid solution of the elements. The solute trapping effect in the AM process implies the existence of the alloying elements in the matrix, and different strengthening effects characterize the resulting solid solution. When the dislocation motion between the grains is suppressed, the hardness of secondary phases is higher than that of magnesium.

Qin et al. [130] have produced AM Zn + xWE43 alloys with a maximum tensile strength of 335.4 MPa and an elongation of 1%. In the Zn + 5WE43 porous substrate, Young’s modulus was found to be 2480 MPa, and the compressive strength was equal to 73.2 MPa. Li et al. [69] developed, using SLM technology, porous WE43 scaffolds with diamond cells. The Young’s modulus was 0.7–0.8 GPa, very close to the value of trabecular bone after 4 weeks of biodegradation. The implant pore size was about 600 μm, and the strut size was 400 μm.

In Table 6 are presented the mechanical properties of SLM-produced Mg-based alloys.

There is little information in the literature regarding SLM-produced Mg alloys and their mechanical properties, which are better than in the case of as-cast magnesium [135,137]. At a laser energy density between 104 and 167 J/mm^3^, the UTS and YS of SLM-produced AZ91 alloy are 30% and 50% higher than values obtained for as-cast AZ91. The elongation is 40% lower compared to values obtained for as-cast materials. For AZ61 alloy at a laser energy density of 156 J/mm^3^, the UTS and YS are equal to 287 MPa and 233 MPa, respectively, which are about 93% and 135% higher than in the case of as-cast material [136]. For this alloy, the elongation rate has an average value of 2.71%, which is lower than the values obtained for as-cast AZ61. A physical explanation of these observations is that the SLM process is directly linked to smaller grain sizes and a uniform microstructure. It was noticed that the UCS and Young’s modulus increase is directly proportional to the laser power energy density [129]. A possible cause can be considered by the material’s different porosities obtained at variable energy inputs. When the alloy porosity decreases, an increase in UCS, plasticity, and elastic modulus is seen.

In addition, an important experimental observation is the fact that the mechanical properties of magnesium exhibit an important anisotropy, and the longitudinal mechanical properties are better than the transverse mechanical properties. Stress concentrations are due to pores, and reduced mechanical properties are expected. It is important to control the pore size in Mg-Ca alloys by modifying the laser energy [129].

### 5.4. Microstructure

The scaffold microstructure is important regarding the physical properties such as toughness, strength, ductility, wear resistance, hardness, and corrosion rate. The AM implant microstructure depends on processing conditions and the chemical composition of the powder material. Due to high cooling rates, a microstructure characterized by much finer grains is obtained using the SLM technique. The Mg-based parts fabricated through AM have an average grain size of 30 μm. They are characterized by a homogenized microstructure that appears during the rapid solidification process, which is characteristic of the SLM method. An improved microstructure is directly connected to better mechanical and biological properties.

**Figure 6:**
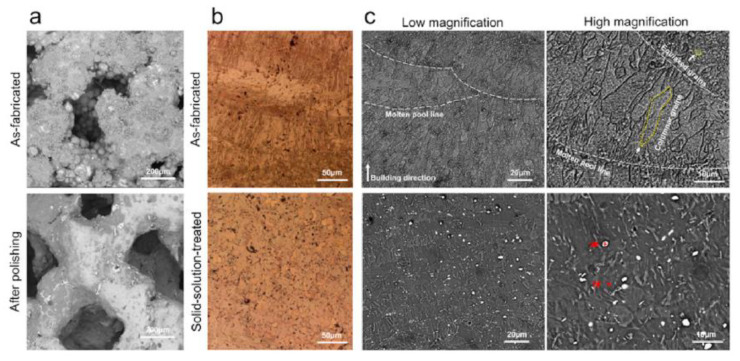
Characterization and degradation behavior of AM-manufactured JDBM scaffolds: (**a**) surface morphology; (**b**) optical microscopy images; (**c**) scanning electron microscopy images. Figure is licensed under CC−BY 4.0 [138].

Xie et al. [138] investigated the microstructure of AM-manufactured scaffolds of Mg-Nd-Zn-Zr (JDBM). In the case of the as-fabricated scaffold, fish-scale-shaped melt pools were observed. These structures are composed of equiaxed and columnar grains. The latter grew along the molten pool, and the equiaxed grains were formed on the boundary of the molten pool. White dot particles were located in the grain interiors (Figure 6). The authors attributed this to the Mg_12_Nd phase induced during the solidification process. After a heat treatment was applied, the columnar grains disappeared. The material contained only equiaxed grains with an average size of about 22.5 μm and white particles. The white dot chemical composition was investigated through X-ray diffraction analysis (XRD), and it was found that some dots represent a rare earth hydride NdH_2_ and Mg_12_Nd eutectic phase.

**Figure 7:**
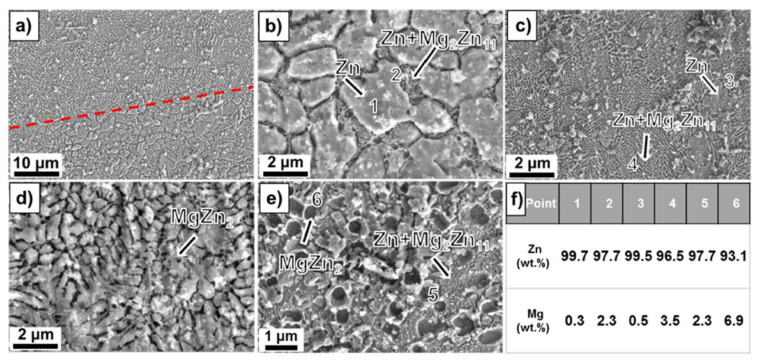
Microstructure analysis: (**a**) and (**b**) Zn-2WE43; (**c**) and (**d**) Zn-5WE43; (**e**) Zn-8WE43, (**f**) element ratio obtained through EDX analysis [130]. Figure is licensed under CC−BY−NC−ND 4.0. Reprinted with permission from ref. [130]. Copyright Elsevier, 2022.

Dong et al. [131] analyzed the microstructure of Mg-Zn alloy scaffolds produced through extrusion-based additive manufacturing. The implants had a porosity of 50.3% and strut density of 93.1% and were composed of a Mg matrix and MgZn_2_ second-phase particles. It was concluded that the etched Mg-Zn samples exhibited a grain size of 26.5 ± 3.5 μm with second-phase particles dispersing at grain boundaries. For the pure Mg specimen, an average grain size of 28.3 ± 1.2 μm with clean grain boundaries was found (Figure 7). The XRD analysis gives evidence in the case of Mg-Zn for the presence of α-Mg phase and MgZn_2_ second phase.

The influence of the Mg content on the microstructure was investigated in [130] for laser powder bed fusion (L-PBF) Zn-Mg alloy porous scaffolds. The porosity of L-PBF samples was found to be in the range of 50–53%. Secondary phases such as Mg_2_Zn_11_ and MgZn_2_ were detected based on X-ray diffraction (XRD) and energy dispersive X-ray (EDX) investigations. Scanning electron microscopy (SEM) gave evidence, in the case of Zn-1Mg, of a polygonal α-Zn phase, and fine α-Zn + Mg_2_Zn_11_ eutectic phases. When the Mg concentration increases, a reduction of the primary α-Zn phase, an increase in the eutectic phase, and the apparition of MgZn_2_ phase dots can be noticed. It was concluded that the increased Mg content is directly linked to a refined grain structure (Figure 7).

L-PBF-manufactured WE43 porous scaffolds composed of 4.26% Y, 2.46% Nd, 1.28% gadolinium (Gd), 0.43%Zr, and residual Mg were investigated in [139]. XRD analysis proves the presence of α-Mg, Y_2_O_3_, and β phase. The flake phases were supposed to be oxides, which were crushed into flakes during the L-PBF process. This structure could not be melted due to experimental conditions, and it was assumed that the dissolved element Y could react with the residual oxygen in the L-PBF chamber. Mg_3_X eutectic compounds precipitated from the liquid WE43, where X represents rare earth (Y, Gd, or Nd). STEM showed the presence of a hybrid oxide of (Y,Zr)_2_O_3_. Secondary phases such as Mg_14_(Nd,Gd)_2_Y along grain boundaries were observed. Li et al. [140] manufactured WE43 Mg alloy scaffolds using the laser powder bed fusion method. The presence of oxide particles and intermetallic precipitates of Mg and rare earth (RE) was noticed in the Mg matrix. The latter are smaller and can be observed in the vicinity of the melt pool boundaries. Electron backscatter diffraction (EBSD) analysis gave evidence of a bi-modal grain size distribution with small grains with random texture located near the melt pool and large grains oriented parallel to the building direction.

### 5.5. Biocompatibility

**Figure 8:**
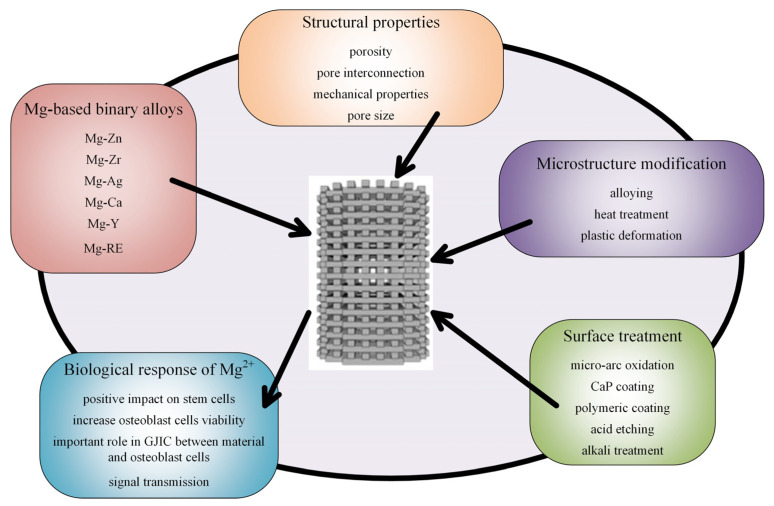
Type of alloys, microstructure modifications, surface treatments, and biological effects of Mg-based scaffolds.

Biocompatibility is a fundamental property of medical implants. The powder composition that is used in the additive manufacturing procedure must be properly designed, and information regarding bulk material could represent a starting point in this process. In order to obtain increased biocompatibility, an improvement of the mechanical properties, and maintain the material integrity for magnesium-based alloys through surface biofunctionalization, microstructure modification and surface treatments have to be considered (Figure 8).

Li et al. [140] investigated open porous scaffolds made from WE43 Mg alloy designed using the laser powder bed fusion technology. A body-center cubic pattern with different strut diameters was produced. The scaffolds’ microstructure was improved with a thermal solution and heat aging treatments. It was noticed that the increase in the strut diameter to up 800 μm generated an increase in the elastic modulus from 0.2 to 0.8 GPa. Additionally, through plasma electrolytic oxidation (PEO) treatments, the material corrosion rate was decreased to approximately 0.1 mm/year, and good biocompatibility was achieved. Wu et al. [141] prepared Mg-6Zn-0.6Zr (ZK60) scaffolds using the selective laser melting (SLM) method. A laser power of 50 W and a scanning velocity of 500–800 mm/s led to minimal defects and high dimensional accuracy samples. It was noticed that SLM ZK60 has a reduced grain size of 7.3 μm in comparison with the 56.4 μm obtained in the case of cast ZK60. On the same consideration, it exhibited a higher hardness of 0.78 GPa and similar values of the elastic modulus. Higher corrosion resistance was identified for SLM ZK60 in Hanks’ solution with a decrease of 30% in hydrogen evolution rate and 50% in the corrosion current density. Yang et al. [68] made bioglass-reinforced Mg-based composite via laser additive manufacturing. The samples were characterized by a refined and homogenized structure, which improves the material corrosion rate. The composite material has good biocompatibility because it promotes cell growth and differentiation, and rapid bone healing was reported. Yao et al. [142] have prepared binary and ternary Mg-based alloys using SLM technology. An improvement of the microhardness was noticed in the case of Mg-0.6Ca and Mg-0.5Zn-0.3Ca, with measured values being approximately equal to 55 HV. These laser-processed magnesium alloys with an improved corrosion rate showed excellent biocompatibility. Xu et al. [143] improved the biodegradation resistance through grain refinement methods in the case of ZK30 + Cu alloys produced via SLM. These types of materials exhibit antibacterial properties due to the copper alloying procedure. When the Cu percent is increased, the Vickers hardness can be up to 98 HV for 0.3 wt% Cu, and the corrosion current is equal to 47.8 μA/cm^2^. These materials exhibited good cytocompatibility and high antibacterial properties against colonies of *S. aureus*. It was observed that an increase in the Cu content is directly linked to a faster decrease in the colonies’ number. Yin et al. [144] studied in vitro degradation behavior and cytocompatibility of ZK30/bioactive glass composites produced through SLM. They have concluded that the integration of bioactive glass (BG) into Mg-based alloys leads to increased corrosion resistance, microhardness, and biocompatibility and that the alloy ZK30/10BG is a promising material for orthopedic applications. Shuai et al. [145] developed an antibacterial Mg-based ZK60-*x*Cu alloy, with *x* between 0.2 and 0.8 wt%, prepared by SLM. Antibacterial properties of the material were analyzed through the bacterial counting method using *Escherichia coli* as a bacterium model. It was observed that the colonies decreased with the increase in the immersion time on ZK60 + Cu alloys. In the case of ZK60-0.6Cu and ZK60-0.8Cu, the colonies disappeared after a specific time. This fact was due to a combination of copper (Cu) ions’ release and an alkaline environment that is beneficial for cellular membrane structure deterioration and bacterial annihilation. Copper can modify enzyme activity and can inhibit deoxyribonucleic acid (DNA) replication. In order to test the alloy’s biocompatibility, human osteosarcoma MG63 cells were used. The reported results showed very good cytocompatibility. Regarding the material microstructure, the alloying process of ZK60 with Cu produced a grain refinement that improved mechanical properties.

## 6. Biological Properties of Magnesium-Based Scaffolds

The standards regulating the interaction between cells and new scaffold materials are ISO 10993-5 and 10993:12. The standardized tests include direct and indirect contact between cells and materials and cytotoxicity tests such as extraction-based assays [146,147]. Unfortunately, the latter were developed for non-degradable implants, and in the case of Mg-based alloys, this method cannot be applied according to the International Organization for Standardization (ISO). A gas release is observed when a Mg scaffold is immersed in the extraction medium. This phenomenon is followed by an increase in the pH value and a strong degradation effect. Sometimes an osmotic shock that kills the living cells can be seen. In order to assess this drawback, in vitro bioreactors that simulate the human body through a dynamic flow system can be involved. Many researchers have undertaken cytotoxicity tests consisting of cell culture medium [148] analyzed under physiological conditions (CO_2_ level of 5%, O_2_ level of 20%, relative medium humidity of 95%, and temperature of 37 °C) such as 10% fetal bovine serum. Dilution of pure extracts can lead to experimental mistakes due to the percentage of Mg ion reduction. The EN ISO standards 10993:5 and 10993:12 recommend that the sample weight to extraction medium ratio be 0.2 g/mL. Fischer et al. [149] conducted experiments to analyze the influence of magnesium extracts on osteosarcoma and human osteoblast cell lines. The specimen samples were prepared from pure magnesium (99.95%), magnesium with 0.6 wt% Ca, and magnesium with 1 wt% Ca using permanent mold direct chill casting. These samples were incubated in Dulbecco’s modified Eagle medium (DMEM) Glutamax-I supplemented with 10% fetal bovine serum. The osmolality of the extract was measured with a Gonotec 030-D cryoscopic osmometer. An increase in osmolality was noticed, and it was concluded that this phenomenon is directly linked to increased Mg concentration. All cytotoxicity tests proved a higher tolerance of the osteoblasts towards Mg extracts compared with the human osteosarcoma cell line. The alloys that contain Ca showed better cell proliferation qualities.

Some authors consider that MTT and XTT assays, which involve tetrazolium salt use, can also provide altered results because degraded Mg reacts with tetrazolium salts, resulting in formazan formation that has a negative impact on in vitro tests [150]. These assays are fast, facile, non-radioactive, and work with metabolically active and living cells. An important disadvantage of this technique is that the tetrazolium-based analysis does not distinguish between cell death and cell reduced growth rate. The tests can be influenced by different substances such as human serum albumin or vitamins C and D that reduce the MTT and XTT tetrazolium salt percent. Other chemicals that influence these assays are flavonoids, thiol-containing antioxidants, D-glucose, nanoparticles, or glutathione S-transferase. Fischer et al. [150] compared the efficiency of MTT and XTT assays with luminescence-based assay (BrdU). Using the permanent mold casting method and dry pressing and sintering technology, pure magnesium, Mg-Y (4 wt% yttrium), and Mg-Ca-1eu (1 wt% calcium, eutectic) samples were prepared. All the samples were sonicated for 20 min in dry isopropanol, and after that, they were gamma sterilized. Regarding the cell culture, they used human osteosarcoma MG63 cultured in Dulbecco’s modified Eagle medium combined with 10% fetal bovine serum. It was concluded that sometimes MTT or XTT assays could not identify the cell viability in the right manner, and the influence of different factors must be considered. An adequate method for Mg-based alloys in the case of cytotoxicity tests is luminescence-based assay (BrdU), which does not interfere with the Mg corrosion process.

For in vitro tests, different cell lines such as fibroblastic, human osteoblast, mouse pre-osteoblastic, or human osteosarcoma cells are used. Unfortunately, in all the investigated cases the osmosis phenomenon can lead to cell apoptosis. Osmotic swelling or shrinkage can have an important influence on the cell proliferation process [70,151]. The interactions between Mg ions and different cell types must be separately analyzed because different cytotoxicity responses can be obtained. Bobe et al. [70] manufactured open porous scaffolds made of sintered Mg-Y (W4) short fibers. The material’s biocompatibility was tested based on material extracts and the mouse fibroblast (L929) cellular line. Ten open porous samples were prepared, and then they were incubated in physiological conditions. Another 10 W4 samples were made following the same protocol; the only difference was that human osteoblast cells (HOB) were used. Weight loss and corrosion rate estimation was performed, and similar viability and proliferation testing for L929 and HOB was carried out. The authors have concluded that the cytotoxicity tests depend on many environmental conditions. The study proved that different cell lines, such as fibroblastic cell lines (L929) and human osteoblast cells (HOB), have different responses to ionic and osmotic modifications. HOB survival rate is higher than L929 in high-concentration osmotic solutions, but cellular proliferation in the case of human lines is much more reduced than that obtained for mouse cell lines when highly osmotic extracts are used. Wu et al. [152] prepared degradable Zn-0.04Mg-2Ag alloy scaffolds for large-scale bone defect treatment. Regarding the cytocompatibility of the scaffold, the authors used MC3T3-E1 mouse pre-osteoblast cells cultured in an α-minimum essential medium (MEM) that contains 10% fetal bovine serum (FBS). The cell suspension had a 50,000 cells/mL density, and 5000 MC3T3-E1 were added to wells in 96-cell plates. The cytoskeleton staining of cultured cells with Zn-0.04Mg-2Ag exhibited an evident outline of cells and nuclei, and the cells infiltrated into the scaffold structure. The proposed implants showed a slight antibacterial effect on *Escherichia coli* and a highly antibacterial effect against *Staphylococcus aureus* and *Staphylococcus epidermis*. The investigated Mg-based alloys proved to up-regulate the mRNA expression for osteoblast-specific transcription factors such as osteopontin and osteocalcin. Wang et al. [55] proposed novel porous Mg-Nd-Zn-Zr coated with brushite and proved that these implants are adequate for cell adhesion, osteogenic differentiation, and bone regeneration of a critical defect surgically induced in the femoral condylar of rats and radius segmental bone defects of rabbits. For in vitro tests, they used rat bone marrow mesenchymal stem cells (rBMSCs) collected from the femoral zone of Sprague Dawley (SD) rats. An α-MEM medium with 10% fetal bovine serum, 100 units/mL penicillin, and 100 units/mL streptomycin was prepared and then incubated for 72 h. Ninety-six-well cell culture plates with a cell density of 5 × 10^4^ cells/mL were cultured with 200μL extract in each well for a time interval between 1 and 7 days. The scaffolds promoted osteogenic differentiation, and enhanced the mineralization process, angiogenesis, and osteogenesis. Xie et al. [138] prepared 3D-printed JDBM scaffolds and analyzed the implant cytocompatibility in clonal murine cell line of immature osteoblasts derived from mice (MC3T3-E1) and murine macrophage (RAW267.4) cells. They cultured the cells in the presence of different extract concentrations. It was concluded that 50, 25, and 12.5% of sample extracts did not inhibit the cell viability, did not increase the number of dead cells, and did not change the cell morphology. Dong et al. [131] manufactured an Mg-Zn alloy scaffold using an extrusion-based manufacturing process. The indirect culture of MC3T3-E1 mouse pre-osteoblasts in Mg-Zn extract proved good cytocompatibility. The MC3T3-E1 cells were pre-cultured for 7 days in α-MEM solution without ascorbic acid and supplemented with 10% FBS and 1% penicillin/streptomycin under physiological conditions. It was observed that the pH values of extracts of Mg-Zn and pure Mg were lower than the pH tolerance threshold of MC3T3-E1 cells. The Mg^2+^ ion release limit for MC3T3-E1 cells is less than 360 mg/L, and it was noticed that in the case of 50% and 100% extracts of Mg-Zn and pure Mg, there were some cytotoxic reactions. High Zn^2+^ concentration had an inhibitory effect on the pre-osteoblasts’ growth. It was concluded that a low Zn^2+^ concentration promotes migration, viability, and proliferation. A safe value for the concentration of Zn^2+^ was reported to be 3 mg/L, and only the 10% Mg-Zn extract was cytocompatible. Qin et al. [75] used the same cell line to test the cytocompatibility of Mg-Zn scaffolds manufactured through laser powder bed fusion (L-PBF). Pure Zn and Zn-1Mg 100% extracts exhibited a high toxicity grade to MC3T3-E1 cells. In the case of Zn-5Mg alloy extracts, the best cell viability was found, and after 5 days, the differences in cell viability between samples decreased.

It is well known that the Mg ions regulate the calcium (Ca) ion level, which has a beneficial effect on cell metabolism and shape. Zhang et al. [153] gave evidence of the importance of Mg ions (Mg^2+^) and the antagonistic effects of Ca^2+^ ions and Mg^2+^ in vascular smooth muscle cells to control vascular reactivity. Mg^2+^ affects the Ca^2+^ flux across vascular muscle membranes and its release from the human body. Lang et al. [154] investigated the cell volume regulatory ion channels in cell proliferation and death. An increase in cell volume characterizes cell proliferation, and apoptosis is directly linked to cell shrinkage. In order to modify the volume of the cell, one must involve ion transport across the cell membrane and an adequate activity of Ca^2+^, Cl^−^, and K^+^ channels. K^+^ exit decreases the cytosolic K^+^ concentration, which can induce the cells’ apoptosis. Ca^2+^ enters through Ca^2+^-permeable cation channels. A hyperosmotic shock activates this process, and an increase in cytosolic Ca^2+^ activates both cell proliferation and apoptosis. Cl^−^ channels have an influence on cytosolic Cl^−^ activity, and it can mediate the osmolyte flux.

In Table 7 are summarized some studies regarding the Mg-based alloy scaffolds’ cytocompatibility, the involved cell lines, and the obtained conclusions.

## 7. In Vivo Behavior of the Magnesium-Based Scaffolds

**Figure 9:**
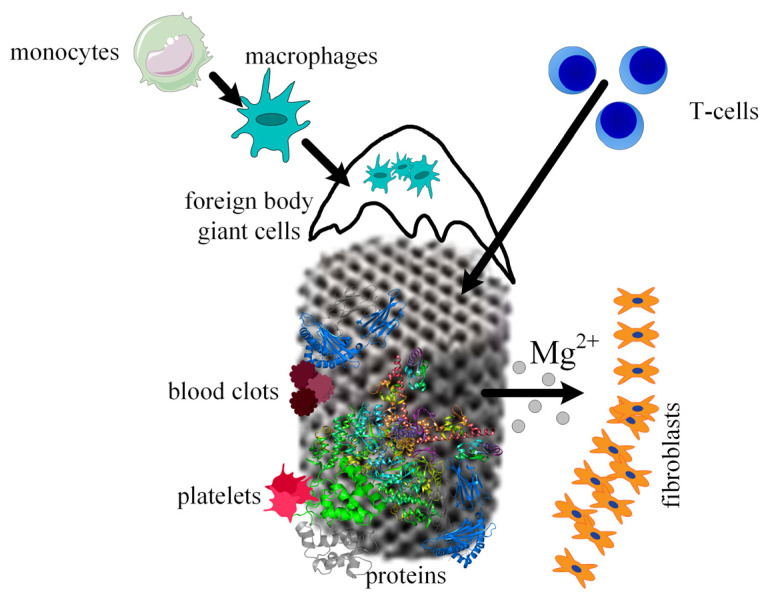
Osseointegration process of the scaffold. Firstly, proteins adhere to the scaffold material, then platelets begin the blood clotting formation, immune system cells migrate to the wounded tissues due to the inflammation process, Mg^2+^ ions diffuse away as a consequence of the metal corrosion, and fibrous tissue encapsulation appears.

When a scaffold is designed for a given medical application, the interaction between host tissue and scaffold materials, the corrosion process of the biodegradable implants, the ingrowth and ongrowth of newly generated tissue combined with the apparition of new blood vessels, and foreign body responses must be taken into consideration. It is well known that scaffold integration in the human body is made up of three steps. The first one appears 2 weeks after surgery and consists of initiating and developing an inflammatory response. This phenomenon is characterized by the apparition of plasma cells, polymorphonuclear leukocytes, lymphocytes, and monocytes. During the second phase, the monocytes become predominant and differentiate into macrophages. New blood vessels and implant encapsulation with fibrous tissue are present. A direct link was observed between the scaffold’s degradation and the time interval of this second step. In addition, foreign body giant cells are seen at the scaffold surface due to the fusion or joining of the macrophage cells. In the third phase, the implant is degraded, losing its mechanical integrity. The scaffold is slowly replaced with new fibrous tissues, which change its structure during a certain time. Finally, the scaffold breaks down into particles, which are eliminated through phagocytosis by the macrophage cells (Figure 9).

Xie et al. [138] manufactured biodegradable porous Mg-Nd-Zn-Zr implants using the SLM technique for scaffold-related infections. The in vivo test consisted of scaffold implantation in a rabbit model. They were proved to have high antibacterial properties against methicillin-resistant *S. aureus* and *Escherichia coli*. The implant biocompatibility was investigated based on blood tests, histological evaluation, and Mg^2+^ deposition measurements. During the first stage of the implantation, inflammatory response and TNF-α secretion were seen at the boundary between the scaffold and rabbit tissue. It was noticed that the high concentration of Mg^2+^ ions promotes the M1 phenotype of macrophages, enhancing their phagocytic ability. In conclusion, it was stated that 3D-printed porous JDBM scaffolds have great potential in the orthopedic field, especially for patients that have a high risk of infections.

Qin et al. [75] developed Zn-Mg alloy porous scaffolds for enhanced osseointegration produced through laser powder bed fusion, using pre-alloyed Zn-xMg powder, with x between 1 wt% and 5 wt%. The in vivo investigation took into account histological analysis after 6-week and 12-week implantation in rabbit femurs. Enhanced bone formation for the Zn-xMg scaffold compared with pure Zn implants was found. It was concluded that this material is promising for large bone defect treatment because it has a high osteogenic effect. The biocompatibility and osteogenesis increase directly proportional to the Mg percent. Excessive addition of Mg produces a decrease in mechanical properties.

WE43 porous scaffolds were fabricated through laser powder bed fusion by Liu et al. [85]. For in vivo tests, they used 45 6-month-old male New Zealand white rabbits with weights between 3 and 3.5 kg. In the left knee of the animal models, a surgical defect with a diameter of 5 × 6 mm^2^ was created. The animals were divided into three groups: for the first one, the defect was left empty; for the second one, WE43 scaffold treatment was applied; and in the case of the third group, calcium sulfate bone cement was used to fill the defect. At 4, 8, and 12 weeks, the rabbits were euthanized, and femur samples were analyzed. The in vivo compatibility was evaluated as a function of the presence or absence of rejection reaction, inflammation, infection, and fester. The animal blood was used to determine alanine transaminase (ALT) levels, UREA, and Mg^2+^ concentrations. It was concluded that the WE43 scaffolds lost mechanical integrity at 4 weeks after implantation. The healing time of the defect was found to be different, and it depended on the clinical conditions. It is well known that clinical studies reported a time between 6 and 12 months for the complete degradation of Mg screws. The gas bubbles resulting due to the Mg corrosion process were visible at 4 weeks, and they completely disappeared after 8 and 12 weeks. Regarding the osseointegration and osteoinduction properties of the WE43 scaffolds, it was noticed that at 12 weeks after the surgery, the defect was filled with new trabecula. Empty cavities were seen in the case of the untreated group and cement group. It was concluded that if the degradation rate of WE43 is improved, the developed scaffolds show high biocompatibility and good osteoinduction properties and can be used in the orthopedic field.

## 8. Challenges of AM for Mg-Based Alloys

The main challenge with additive manufacturing of Mg alloys consists of powder preparation because Mg oxidizes and because, in the case of powder, the surface energy rises; a burning effect can appear when the material interacts with the air. In order to address this drawback, an inert atmosphere and specialized equipment will be necessary [155]. Mg powders are produced through evaporation–condensation, mechanical crushing, electrolytic methods, and water and gas atomization [156]. In the case of the last method, the optimal Mg particle size, which can be used in the case of additive manufacturing techniques, is between 20 μm and 70 μm. The material properties could be severely altered when gas infiltrates the Mg powder. The particle size influence was investigated in the literature, and it was shown that a decrease in this parameter is directly linked to a lower porosity and a high material density [157]. It was noticed that powders prepared using the gas atomization method exhibited higher relative density than water-atomized particles because of the differences between the two technologies regarding flowability, oxygen contents, packing density, and particle morphology.

Another important challenge is the powder spatter, which can be identified during the AM procedure. The vapors can remove Mg powder particles along the scanning path, so as a consequence, the defects’ apparition is favored. In order to assess this drawback, a powder supplement step can be added.

In the case of Mg cubes manufactured through AM, some cracks can appear [104,137]. It is considered that the defect source is linked to the powder splash phenomenon, which decreases under reduced energy input. The variation and complexity in the thermal cycles that characterize the PBF process make it very difficult to fabricate samples with minimum porosity grade and cracks. Regarding the SLM technology, a local laser energy input source is used to melt and cool the powder rapidly. Due to a high-temperature gradient, an increase in the residual thermal stress is observed. This fact can contribute to crack formation in the fabricated component.

Cyclic loading combined with the corrosion process led to the so-called corrosion fatigue (CF), which can affect a large number of medical implants. Raman et al. [158] have investigated the susceptibility of magnesium implants to CF. It was noticed that the cracking of Mg-based alloys is strongly influenced by the alloying elements. Aluminum improves corrosion resistance, and Zn produces a strengthening of the solid solution of the alloys. Unfortunately, Al-containing Mg alloys are reported to cause neurological disorders such as dementia or Alzheimer’s disease [39,47]. Recently, in vivo or in vitro studies proved that the Al ions’ release is within the tolerance limit for implant applications. The most used alloying elements involved in medical practice are Ca, Zn, and rare earth (RE). Ca-Mg alloys develop a hydroxy apatite surface layer that improves implant biocompatibility. A grain size refinement and an improvement of the mechanical properties and corrosion resistance are reported when Ca is used as an alloying element. Zinc determines the solid strengthening of Mg, but when a certain percentage higher than 6.2 wt% is reached, Mg-Zn precipitates form, and material embrittlement increases. Rare earth metals improve the creep and corrosion resistance of the alloy because they form fine and stable intermetallic precipitates. One of the most promising materials is Mg-Zn-Ca-Y, which is characterized by good strength and corrosion resistance and can be successfully used as temporary implant material. Metallic contaminants such as Fe, Ni, Co, or Cu can be sources of severe galvanic corrosion because they exhibit a highly cathodic effect on the base alloy matrix phase.

Much more research has to be devoted to the stress corrosion cracking (SCC) resistance of Mg-based alloys. SCC may have as a principal cause the meeting of three variables: the susceptible nature of the alloy, the existence of mechanical loading, which generates tensile stress, and an environment that determines the apparition of the corrosion phenomenon. The implant surface can be unaffected by SCC, exhibiting only a few cracks, which contribute to brittle failure of the implant.

Mg-based alloys exhibit low ductility and are prone to fracture. Due to their low boiling point, the evaporation tendency of Mg increases during SLM methods.

## 9. Conclusions

Additive manufacturing looks to be the proper method to manufacture magnesium-based scaffolds. The most adequate technology is the powder bed fusion that includes no sacrificial material use, such as binders, involved in the binder jetting method or infiltration polymeric templates for indirect AM. Most authors consider that EBM is unsuitable for Mg-based scaffolds’ manufacture due to the Mg evaporation process that interferes with the electron beam in the build chamber. Scaffolds produced through SLM technology are characterized by superior properties such as the absence of voids and high densification. Due to the high cooling rate and solidification, refined grains enhanced solid solution, and homogenized phase distribution materials are obtained using SLM. The development of a novel fabrication technique incorporating a three-dimensional model with a change in porosity across the scaffold volume determines a superior Mg-based implant, enhancing bone/tissue growth.

Regarding the Mg-based scaffolds manufactured through AM technologies, more research for comparative investigation of pore porosity, lattice structure, and topology must be carried out to obtain an ideal scaffold with the best clinical performances. Differences in the biological properties determined for in vivo or in vitro conditions for Mg-based alloys were reported in the literature. By analyzing the in vivo studies, it is seen that there is much more need for research because few studies have investigated this topic. It is essential to establish the period required for the healing of surgically generated bone defects, so that Mg-based scaffolds produced through AM methods can be introduced in clinical trials.

A significant problem when magnesium powder is used consists of the pyrophoric character of the material. As it exhibits a high-energy surface, the printing of biodegradable Mg-based implants must be performed in an inert atmosphere, and the process is challenging and must be carefully checked.

A major concern identified in the literature [15] is that Mg-based alloys, when put in contact with a physiological medium inside the human body, release byproducts such as hydrogen or hydroxyl ions. Lately, porous Mg-based implants have been used more and more as a biodegradable cancellous bone replacement due to their high biocompatibility and adequate mechanical properties [159]. The chemical composition or microstructure modification can be carried out by alloying the Mg-based materials or by different surface modifications that can be performed before the implantation of the scaffolds [160,161]. Some studies stated that through Zn incorporation of less than 3%, the mechanical properties of the scaffolds [162] and the corrosion resistance [163] increase. When a small amount of alloying materials such as Mn, Ca, Sr, Zr, Si, Li, or rare earth metals is added, the release of hydrogen can be hindered, but if this quantity is increased, the excessive element addition can harm the corrosion properties of the material. Surface modification such as surface coating preparation through chemical conversion coatings, biomimetic deposition, microarc oxidation coating, sol–gel coating, and ion implantation, and surface microstructural modification based on mechanical attrition, shot peening, laser, and friction stir processing can be successfully applied to reduce the corrosion byproducts of Mg-based alloys [164]. Other proposed solutions include magnesium hydroxide formation and hydrogen bubbles’ release in the electrolytic physiological environment after the alloy degradation begins [39,165].

A major limitation of the many studies found in the literature is that the Mg-based scaffold structure is not populated with different cell types to analyze the cells’ stability and proliferation in the Mg-based scaffold structure. This research direction must be addressed soon, together with the correlation between scaffold porosity and cell dimensions, as well as interaction between scaffolds and biological factors. Simultaneous printing of living cells and metals has encountered problems due to incompatible printing conditions. On the other hand, feasibility studies regarding the heat transfer characteristics to integrate biological moieties within biodegradable metallic structures that can open innovative and new research directions are necessary.

## Figures and Tables

**Table 1:** Materials used in bone tissue engineering applications.

Material	Type	Remarks	Reference
Nickel–titanium alloy (NiTi)	Metallic alloy	Even in a porous state, it exhibits a shape memory effect, high biocompatibility, high damping properties, and superplasticity	[20]
Titanium and its alloys (Ti)	Metal	Inert and is capable of osseointegration with bone, has superior biocompatibility, and has good mechanical properties	[21]
Magnesium (Mg)	Metal	Fully bioresorbable, high biocompatibility, good mechanical properties, and high osteoconductivity. It induces no inflammatory responses	[22,23]
Porous tantalum (Ta)	Metal	High volume porosity (>80%), interconnected pores, modulus of elasticity similar to that of bone	[24]
Bioactive glass (BG)	Ceramic	Antibacterial properties, low fracture toughness	[25,26]
Tricalcium phosphate (TCP)	Ceramic	High biodegradability and solubility, increased biocompatibility, low mechanical properties	[27,28]
Hydroxyapatite (HA)	Ceramic	Biocompatible, highly osteoconductive, not suitable to be used as a stand-alone supportive scaffold	[29]
Poly lactic-co-glycolic acid (PLGA)	Synthetic polymer	Controllable biodegradation property	[16]
Polylactic acid (PLA)	Synthetic polymer	High biodegradability and biocompatibility, controllable geometry	[16]
Polycaprolactone (PCL)	Synthetic polymer	High biocompatibility, easy to manipulate	[30]
Chitosan	Natural polymer	High osteoconductivity and antibacterial properties	[31]
Collagen	Main structural protein in the ECM	High biodegradability, it improves the scaffolds’ biocompatibility	[32]
Silk	Natural protein fiber	Strong fiber, controllable degradation, very easy to process	[33]

**Table 2:** Prestigious journals indexed in Web of Science Core Collection from 2013−2022 having papers on magnesium-based scaffolds.

Number of Published Papers	Journal	Publishing House	Impact Factor
28	Materials Science Engineering C Materials for Biological Applications	Elsevier	8.457
26	Acta Biomaterialia	Elsevier	10.633
22	Materials	MDPI	3.748
16	Journal of the Mechanical Behavior of Biomedical Materials	Elsevier	4.042
15	Eurointervention	Europa Edition	7.728
13	Journal of Materials Chemistry B	Royal Society of Chemistry	7.571
12	Scientific Reports	Nature	4.997
10	Frontiers in Bioengineering and Biotechnology	Frontiers Media	6.064
10	Materials Letters	Elsevier	3.574
9	Bioactive Materials	Elsevier	16.874
9	Biomedical Materials	IOP Publishing	4.103
8	Tissue Engineering Part A	Mary Ann Liebert	4.08
7	Biomaterials	Elsevier	15.304
7	Materials & Design	Elsevier	9.417
5	Polymers	MDPI	4.967

**Table 3:** Advantages and disadvantages of the AM technology used for Mg-based scaffolds’ manufacture.

Method	Advantages	Disadvantages	Examples of Commercially Available 3D Printing Systems	Reference
Selective laser melting (SLM)	Regarding the material, if SLM is used there is no distinction between binder and melting phases. The method is characterized by the elimination of time-consuming and costly furnaces that are used as post-treatments for debinding, post-sintering, and infiltration. SLM is suitable for fully dense parts’ production in a direct way.	The method is not suitable for controlled composite materials. The method requires high laser power, good beam quality, and smaller scanning velocity. SLM exhibits melt pool instabilities and higher residual stresses.	Compact SLM machine BLT S210, China	[84,85,86,87]
Binder jetting	Binder jetting is an economical process for a wide range of part quantities. It does not require printing support. The parts have a good surface quality and dimensional precision, and they are characterized by microstructure homogeneity.	Metal binder jetting needs substantial investments because special binders are used to glue the powder particles. The parts have low mechanical properties, and supplementary treatments for densification are necessary.	Spectrum Z510 printer (Z-Corporation, Burlington, USA)	[88,89,90]
Selective laser sintering (SLS)	SLS does not need support structures. There can be printed components with pronounced details. This fact offers part designers a high degree of design freedom. The method is very fast and exhibits excellent layer adhesion. SLS printed parts have isotropic mechanical properties, so hardness, tensile strength, and elongation have the same value in any spatial direction. The components are ideal for biological treatment in regenerative medicine.	The part can be porous and brittle and prone to shrinkage and warping. The cleaning process of SLS is very difficult due to its specific construction and powders. The technology produces much waste, and it is expensive.	Fuse 1+ 30W SLS 3D printer (Formlabs Inc., Somerville, MA, USA)	[91,92,93]
Indirect additive manufacturing	Mg powder’s use is eliminated due to the infiltration method used in this technology.	The parts have pores and struts limited to macroscale, which are inadequate for medical use.	Mold fabrication (ProJet 3000, 3DSystems, USA); Mixer (Caframo RZR2-64, Canada); Induction furnace (Induktio, Slovenia)	[94]

**Table 4:** Biodegradation property of AM Mg-based scaffolds.

Mg Alloy	Geometry	Biodegradation Behavior	Reference
Mg-4Y-3RE-0.5Zr (WE43)	Scaffold with diamond lattice porous cylinder	Biodegradation behavior is characterized by around 20% volume loss after four weeks	[69]
Mg-6Zn-0.6Zr (ZK60)	Non-porous block	Hydrogen evolution rate was investigated in Hanks’ solution (0.006–0.019 mL cm^−2^ h^−1^)	[126]
Mg-xZn	Non-porous block	Hydrogen evolution rate was investigated for Mg-6Zn, Mg-2Zn, Mg-4Zn, and Mg-8Zn	[100]

**Table 5:** Formation quality of additively manufactured Mg-based porous materials and scaffolds.

Material	Formation Quality	Topology	Reference
Mg-Ca	Average densification of 78.46%	Porous structure	[129]
Mg-Zn-Zr	Relative density of 97.4% at a laser energy of 600 J/mm^3^	-	[126]
Mg-4Y-3RE-0.5Zr (WE43)	Geometrical discrepancies regarding a higher value of the as-built strut size and a lower value of the as-built porosity than in the designed case	Diamond lattice porous scaffold	[69]
Zn + WE43	An average relative density of 99.7%. Geometrical discrepancies regarding the strut size and porosity	Diamond lattice porous scaffold	[130]
Mg-Zn	An average strut width of 581.2 ± 14.9 and an absolute porosity of 58.3 ± 3.4%	Cylindrical porous structure	[131]

**Table 6:** Mechanical properties of some SLM-manufactured Mg-based alloys.

Alloy	Energy Density [J/mm^3^]	Ultimate Tensile/Compressive Strength (MPa) UTS/UCS	Yield Strength (YS) (MPa)	Elongation (%)	Reference
Mg-9Al-1Zn (AZ91)	104–167	296–330 UTS	254–264	1.24–1.83	[135]
Mg-6Al-1Zn (AZ61)	156	287 UTS	233	3.28–2.14	[136,137]
Mg-Ca	625–1125	5–46 UCS horizontal/51–111 UCS longitudinal	-	-	[129]

**Table 7:** Mg-based scaffolds’ cytocompatibility.

Mg-Based Alloy	Cell Line	Conclusions	Reference
Pure magnesium (99.95%), magnesium with 0.6 wt% Ca, and magnesium with 1 wt% Ca	Osteosarcoma and human osteoblasts	All cytotoxicity tests proved a higher tolerance of the osteoblasts towards Mg extracts compared with the human osteosarcoma cell line. The alloys that contain Ca showed better cell proliferation qualities	[149]
Pure magnesium, Mg4Y (4 wt% yttrium), and MgCa1eu (1 wt% calcium, eutectic)	Human osteosarcoma MG63	Sometimes MTT or XTT assays could not identify the cell viability in the right manner, and the influence of different factors must be considered. An adequate method for Mg-based alloys in the case of cytotoxicity tests is luminescence-based assay (BrdU), which does not interfere with the Mg corrosion process	[150]
Mg-Y (W4) short fibers	Mouse fibroblasts (L929) and human osteoblast cells (HOB)	HOB survival rate is higher than L929 in high-concentration osmotic solutions, but cellular proliferation in the case of human lines is much more reduced than that obtained for mouse cell lines when highly osmotic extracts are used	[70]
Zn-0.04Mg-2Ag alloy	MC3T3-E1 mouse pre-osteoblast cells	The investigated Mg-based alloys proved to up-regulate the mRNA expression for osteoblast-specific transcription factors such as osteopontin and osteocalcin	[152]
Mg-Nd-Zn-Zr coated with brushite	Rat bone marrow mesenchymal stem cells (rBMSCs) collected from the femoral zone of Sprague Dawley (SD) rats	The scaffolds promoted osteogenic differentiation, enhanced mineralization process, angiogenesis, and osteogenesis	[55]
(Mg-Nd-Zn-Zr) JDBM	MC3T3-E1 and RAW267.4 cells	The scaffolds did not inhibit the cell viability, did not increase the number of dead cells, and did not change the cell morphology	[138]
Mg-Zn alloy	MC3T3-E1 mouse pre-osteoblast cells	It was concluded that a low Zn^2+^ concentration promotes migration, viability, and proliferation. A safe value for the concentration of Zn^2+^ was reported to be 3 mg/L, and only the 10% Mg-Zn extract was cytocompatible	[131]

## Data Availability

Not applicable.

## Abbreviations and Acronyms

3D	Three-dimensional
ALT	Transaminase level
AM	Additive manufacturing
ASTM	American Society for Testing and Materials
AZ31	Mg-3Al-1Zn alloy
AZ61	Mg-6Al-1Zn alloy
AZ91	Mg-9Al-1Zn alloy
BG	Bioactive glass
BrdU	Luminescence-based assay
BTE	Bone tissue engineering
CAD	Computer-aided design
DED	Directed energy deposition
DMEM	Dulbecco’s modified Eagle medium
DNA	Deoxyribonucleic acid
EBM	Electron-beam melting
EBSD	Electron backscatter diffraction
ECM	Extracellular matrix
EDX	Energy dispersive X-ray analysis
FBS	Fetal bovine serum
HAp	Hydroxyapatite
HAZ	Heat-affected zone
HOB	Human osteoblast cells
ISO	International Organization for Standardization
JDBM	Mg-Nd-Zn-Zr alloy
L929	Mouse fibroblast cells
L-PBF	Laser powder bed fusion
MC3T3-E1	Pre-osteoblast cell line from mouse calvaria
MEM	Minimum essential medium
MG63	Human osteosarcoma cells
Mg-CS/PCL	Magnesium–calcium silicate/poly-ε-caprolactone
PBF	Powder bed fusion
PCL	Polycaprolactone
PEO	Plasma electrolytic oxidation
PLA	Polylactic acid
PLGA	Poly lactic-co-glycolic acid
RAW267.4	Murine macrophage cells
rBMSCs	Rat bone marrow mesenchymal stem cells
RE	Rare earth
SCC	Stress corrosion cracking
SD	Sprague Dawley rats
SEM	Scanning electron microscopy
SFF	Solid free-form fabrication
SLM	Selective laser melting
SLS	Selective laser sintering
TCP	Tricalcium phosphate
TE	Tissue engineering
UCS	Ultimate compressive strength
UTS	Ultimate tensile strength
W4	Mg-Y alloy
WE43	Mg-4Y-3RE-0.5Zr alloy
WoS	Web of Science
XRD	X-ray diffraction analysis
YS	Yield strength
ZK30	Mg-3Zn-0.6Zr alloy
ZK60	Mg-6Zn-0.6Zr alloy
